# Chiral Thioxanthones as Modulators of P-glycoprotein: Synthesis and Enantioselectivity Studies

**DOI:** 10.3390/molecules23030626

**Published:** 2018-03-10

**Authors:** Ana Lopes, Eva Martins, Renata Silva, Madalena M. M. Pinto, Fernando Remião, Emília Sousa, Carla Fernandes

**Affiliations:** 1 Laboratory of Organic and Pharmaceutical Chemistry, Department of Chemical Sciences, Faculty of Pharmacy, University of Porto, Rua Jorge Viterbo Ferreira 228, 4050-313 Porto, Portugal; up201402466@ff.up.pt (A.L.); madalena@ff.up.pt (M.M.M.P.); cfernandes@ff.up.pt (C.F.); 2 REQUIMTE, Laboratory of Toxicology, Department of Biological Sciences, FFUP – Faculty of Pharmacy, University of Porto, Rua de Jorge Viterbo Ferreira, 228, 4050-313 Porto, Portugal; up201204736@fc.up.pt (E.M.); remiao@ff.up.pt (F.R.); 3 Interdisciplinary Centre of Marine and Environmental Research, University of Porto, Terminal de Cruzeiros do Porto de Leixões, Av. General Norton de Matos s/n, 4450-208 Matosinhos, Portugal

**Keywords:** P-glycoprotein, thioxanthones, enantioselectivity, expression, activation

## Abstract

Recently, thioxanthone derivatives were found to protect cells against toxic P-glycoprotein (P-gp) substrates, acting as potent inducers/activators of this efflux pump. The study of new P-gp chiral modulators produced from thioxanthone derivatives could clarify the enantioselectivity of this ABC transporter towards this new class of modulators. The aim of this study was to evaluate the P-gp modulatory ability of four enantiomeric pairs of new synthesized chiral aminated thioxanthones (ATxs) **1**–**8**, studying the influence of the stereochemistry on P-gp induction/ activation in cultured Caco-2 cells. The data displayed that all the tested compounds (at 20 μM) significantly decreased the intracellular accumulation of a P-gp fluorescent substrate (rhodamine 123) when incubated simultaneously for 60 min, demonstrating an increased activity of the efflux, when compared to control cells. Additionally, all of them except ATx **3** (+), caused similar results when the accumulation of the P-gp fluorescent substrate was evaluated after pre-incubating cells with the test compounds for 24 h, significantly reducing the rhodamine 123 intracellular accumulation as a result of a significant increase in P-gp activity. However, ATx **2** (−) was the only derivative that, after 24 h of incubation, significantly increased P-gp expression. These results demonstrated a significantly increased P-gp activity, even without an increase in P-gp expression. Therefore, ATxs **1**–**8** were shown to behave as P-gp activators. Furthermore, no significant differences were detected in the activity of the protein when comparing the enantiomeric pairs. Nevertheless, ATx **2** (−) modulates P-gp expression differently from its enantiomer, ATx **1** (+). These results disclosed new activators and inducers of P-gp and highlight the existence of enantioselectivity in the induction mechanism.

## 1. Introduction

P-glycoprotein (P-gp) is the best characterized efflux transporter belonging to the superfamily of ATP-binding cassette (ABC) transporters [1,2,3,4]. The structure of P-gp complies with the prototype characteristics described for the ABC transporters, being a polypeptide made up of two homologous halves that arise from a gene duplication event [5,6,7]. Each half contains a transmembrane domain (TMD), formed of six membrane-spanning α helices (TMHs), and a nucleotide binding domain (NBD), located at the cytoplasmic side of the membrane [4,8,9,10]. This 170 kDa protein, also known as ABCB1, is encoded in humans by the multidrug resistance (MDR) genes *MDR1* (*ABCB1*) and *MDR3* (*ABCB4*), being the multidrug resistance (MDR) phenotype associated with the ABCB1 isoform. Also, the term P-gp refers, usually, to the ABCB1 isoform [4,5,10].

P-gp is broadly expressed in normal tissues, as well as in brain, liver, small intestine, kidney and lung [2,11,12]. Using the energy from ATP hydrolysis, the P-gp promotes the outward transport of a large range of structurally unconnected compounds [4,7,13]. Therefore, it plays important physiological functions, being its primary function to protect the cells against toxic xenobiotics and endogenous metabolites [1,7]. Furthermore, this efflux pump plays a key role in the absorption, elimination, disposition and toxicity of a wide range of compounds, both endobiotics and xenobiotics, since it is responsible for their transport across cell membranes [14,15]. Additionally, it is important to maintain the barrier function of numerous tissues, such as the small intestine, blood–brain barrier and placenta [4,7,9,14,16]. Considering that many drugs used in clinical treatments are substrates of P-gp, their pharmacokinetic processes and, consequently, their effectiveness, can be greatly affected by the level of expression and functionality of this pump [4,7,16,17]. When a drug that is a P-gp substrate is co-administrated with another drug, a P-gp inducer, activator or inhibitor, the pharmacokinetics and bioavailability of the P-gp substrate may be substantially altered [16,18].

Over the years, P-gp modulation has been seen like a vital area in drug development [4,9,11,19,20,21,22]. In fact, due to the P-gp role in MDR, the first studies on this efflux pump were mostly focused on its inhibition as a therapeutic option to circumvent MDR in chemotherapy [3,8,11,22,23]. Since the degree of MDR is strongly correlated with changes in drug permeability, it is largely related to both P-gp expression and activity [21,24]. However, more recent insights showed that P-gp inhibitors can be also helpful to modulate the general pharmacokinetic behavior of drugs in the organism, revealing special importance in case of central nervous system (CNS) active drugs, to allow increasing drugs brain penetration [19,23,25]. Consequently, over the years, several studies were developed in order to discover potent and safe P-gp inhibitors, and to better understand their mechanisms of inhibition [3,23,25,26,27,28,29]. Furthermore, some recent studies have also focused on the induction/activation of this pump, and the use of such inducers/activators to avoid the toxicity mediated by substrates of P-gp has been proposed as a potential antidotal pathway [30,31,32,33]. Accordingly, the modulation of P-gp can be applied not only in drug discovery but also in drug development, to overcome the limitations of some drugs in clinical use due their interaction with P-gp, which affects their clinical effectiveness [4,13,14,29,34].

A group of studied compounds that interact with P-gp activity is the thioxanthones, synthetic *S*-heterocycle compounds with a dibenzo-γ-thiopyrone scaffold. The biological interest in this class of compounds started with lucanthone, firstly introduced as an antischistosomal drug [35,36]. Over the years, diverse biological activities of thioxanthone derivatives have been described, such as P-gp modulation [21,30,37], topoisomerase inhibition [38,39], antitumor [40,41,42,43,44,45,46], and antimicrobial properties [40,41,42,43], among others [47,48,49]. A large amount of studies concerning P-gp modulation by thioxanthones was focused on their ability to act both as P-gp inhibitors and as antitumor agents [11,13,23,25,50,51]. In these studies, some P-gp activators/inducers were disclosed [25]. Indeed, using a Caco-2 cells in vitro model, some thioxanthone derivatives were studied with the aim of evaluate their potential to increase the expression and/or activity of P-gp, and their potential protective effects in Caco-2 cells to fight the toxicity induced by paraquat (PQ) [30]. PQ is an extremely toxic herbicide that is a P-gp substrate, and was used as a model in order to develop new antidotes using this efficient antidotal pathway [30,32,33,52].

There are studies describing that P-gp can interact in a different way with enantiomers [53]. When chiral drugs modulate P-gp, one enantiomer can increase the activity of P-gp while the other enantiomer can inhibit the activity of the pump [24,54]. An example of this type of enantioselectivity was observed for mefloquine, a drug used in the prevention and treatment of malaria: (11*R*,12*S*)-(+)-mefloquine presents a much higher human brain penetration than (11*S*,12*R*)-(−)-mefloquine [55].

**Figure 1:**
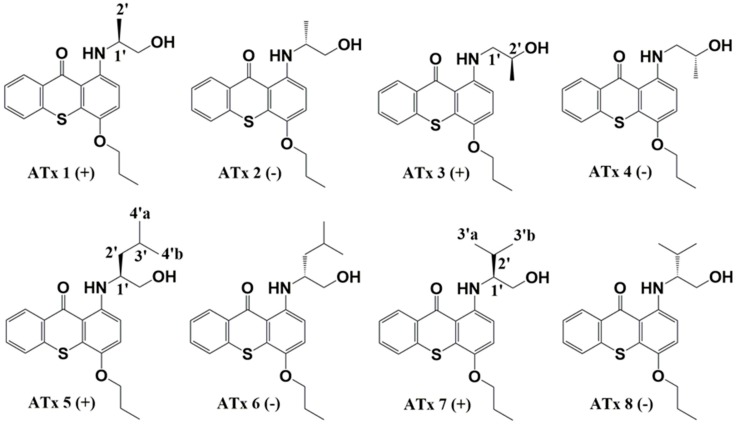
Structures of the studied chiral aminated thioxanthones, ATxs **1**–**8**.

Herein, we investigated the potential modulatory effect on P-gp activity and expression of four newly synthetized pairs of enantiomers of aminated thioxanthones (ATxs **1**–**8**, Figure 1), to clarify the enantioselectivity of this efflux pump towards the ABC transporters class. Moreover, although some examples of biological enantioselectivity were reported in the literature for structurally related xanthones [56,57,58], for thioxanthones, and specifically for P-gp modulation, data is presented here for the first time.

## 2. Results

### 2.1. Synthesis of Thioxanthones

**Scheme 1:**
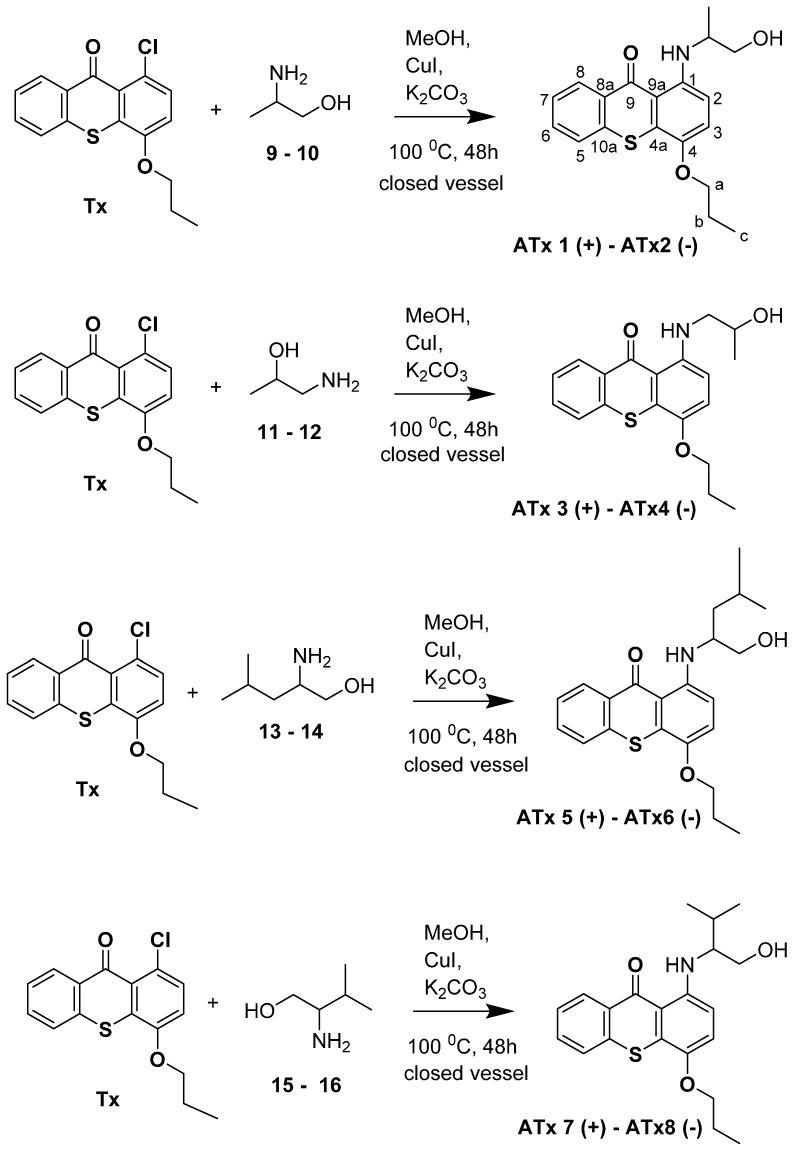
Reaction conditions for the synthesis of chiral thioxanthones **1**–**8** (ATxs **1**–**8**).

The new chiral ATxs **1**–**8** were synthesized by copper-catalyzed Ullmann cross-coupling reactions between the thioxanthone derivative 1-chloro-4-propoxy-9*H*-thioxanthen-9-one (Tx) and eight enantiomerically pure amino alcohols **9**–**16** in alkaline medium (Scheme 1). The amino alcohols selected for this study fitted on the pharmacophore model previously built for P-gp activators and their design was based on a hit compound found as a P-gp activator/inducer, 1-[(3-hydroxypropyl)amino]-4-propoxy-9*H*-thioxanthen-9-one, a thioxanthone with an amino alcohol chain at position 1 [30]. Therefore, we decided to modify this amino alcohol chain introducing also a stereogenic center to explore the influence of chirality in this class of derivatives.

Initial investigations of the catalyst to be used in the Ullman asymmetric reactions revealed copper iodide as a more efficient catalyst when compared to copper oxide used previously in the synthesis of aminated thioxanthones [30].

**Figure 2:**
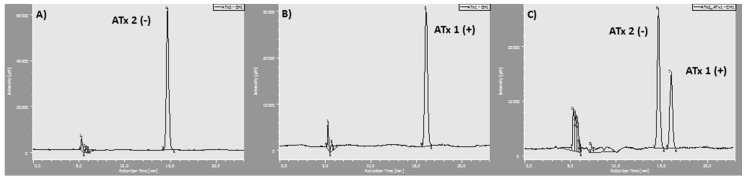
Chromatograms of the investigated compounds (**A**) (*R*)-1-((1-hydroxypropan-2-yl)amino)-4-propoxy-9*H*-thioxanthen-9-one (ATx **2** (−)), (**B**) (*S*)-1-((1-hydroxypropan-2-yl)amino)-4-propoxy-9*H*-thioxanthen-9-one (ATx **1** (+)), (**C**) (*S*,*R*)-1-((1-hydroxypropan-2-yl)amino)-4-propoxy-9*H*-thioxanthen-9-one (mixture of ATx **1** (+) and ATx **2** (−)) on Lux^®^ 5 µm amylose-1 at 0.5 mL/min^−1^; *n*-hexane:ethanol (70:30 *v*/*v*), 0.5 mL/min, λ_max_ 254 nm.

The purity of each synthesized chiral thioxanthone was determined by high performance liquid chromatography with diode-array detection (HPLC–DAD) analysis. All tested compounds presented a purity of at least 95%. Their enantiomeric purity was also measured by chiral HPLC using an amylose-derived column under normal-phase or polar organic mode, achieving enantiomeric excess (e.e.) values higher than 99%, as exemplified in Figure 2 for the enantiomeric pair ATx **1** (+) and ATx **2** (−).

The structure elucidation of all new thioxanthonic derivatives (ATxs **1**–**8**) was established by spectroscopic methods: infrared (IR), ^1^H- and ^13^C-nuclear magnetic resonance (NMR), and bidimensional heteronuclear single quantum correlation (HSQC) and heteronuclear multiple bond correlation (HMBC). Since these determinations were performed in achiral environments, no significant differences were noted for each pair of enantiomers obtained, as expected. The ^13^C-NMR assignments were made by HSQC and HMBC experiments and by comparison with assignments of similar molecules [25,58]. The HMBC data were important to deduce the chemical shifts of quaternary carbons and confirm the C–N coupling product.

### 2.2. Chiral Thioxanthones Cytotoxicity Assays 

**Figure 3:**
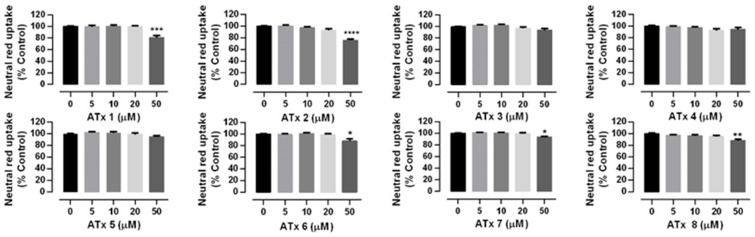
Chiral thioxanthones ATxs **1**–**8** (0–50 μM) cytotoxicity in Caco-2 cells evaluated by the Neutral Red uptake assay, 24 h after exposure. Results are presented as mean ± standard error mean (SEM) from at least 5 independent experiments (performed in triplicate). Statistical comparisons were estimated using the nonparametric method of Kruskal–Wallis (one-way ANOVA on ranks), followed by the Dunn’s multiple comparisons post hoc test (* *p* < 0.05; ** *p* < 0.01; *** *p* < 0.001; **** *p* < 0.0001 vs. control (0 μM)).

Chiral ATxs **1**–**8** cytotoxicity was evaluated by the neutral red (NR) uptake assay, in order to select a noncytotoxic working concentration to be used in the subsequent studies that aim to evaluate their potential to induce and/or activate P-gp. After 24 h of incubation, significant cytotoxicity was observed for the 50 μM concentration of chiral ATxs **1** (+), **2** (−), **6** (−), **7** (+) and **8** (−) (Figure 3). For ATxs **3** (+), **4** (−), and **5** (+) no significant cytotoxicity was observed after 24 h of incubation within the tested concentration range (0–50 μM). Therefore, chiral thioxanthones effect on P-gp expression and activity was further evaluated using a noncytotoxic concentration of 20 μM.

### 2.3. Flow Cytometry Analysis of P-gp Expression

**Figure 4:**
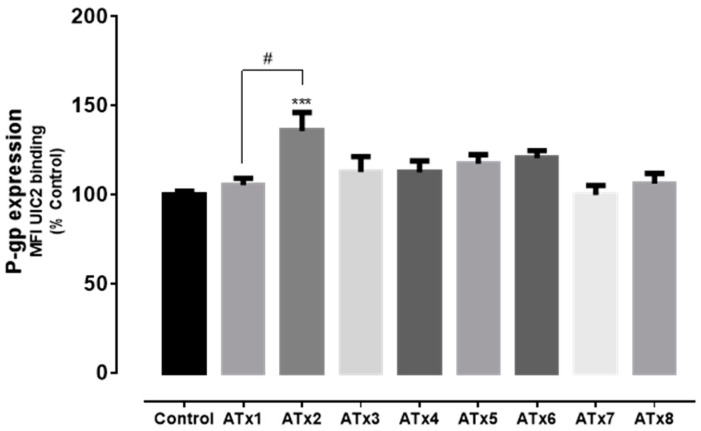
Flow cytometry analysis of P-gp expression levels in Caco-2 cells exposed to ATxs **1**–**8** (20 µM) for 24 h. Results are presented as mean ± standard error mean (SEM) from 5 independent experiments (performed in duplicate). Statistical comparisons were made using One-way ANOVA, followed by Tukey’s multiple comparisons test (*** *p* < 0.001 vs. control (0 µM); # *p* < 0.05 for ATx **1** (+) vs. ATx **2** (−)).

The ability of the tested chiral ATx’s **1**–**8** to induce P-gp expression in Caco-2 cells was evaluated by flow cytometry, using a P-gp monoclonal antibody (UIC2) conjugated with phycoerythrin (PE), 24 h after exposure. As shown in Figure 4, ATx **2** (−) significantly increased P-gp expression by 36%, when compared to control cells. For the other tested chiral thioxanthones, no significant effect on P-gp expression was observed after 24 h of incubation. Furthermore, significant differences were detected in the P-gp induction mediated by the enantiomeric pair ATx **1** (+) : ATx **2** (−), demonstrating an enantioselectivity for P-gp induction.

### 2.4. Evaluation of P-gp Transport Activity 

The P-gp transport activity was evaluated by flow cytometry using rhodamine (RHO) 123, a P-gp fluorescent substrate [25,31]. Two different approaches were used. In the first approach, RHO 123 accumulation was evaluated in the presence of the tested chiral ATxs **1**–**8** (20 μM) during the RHO 123 accumulation phase that lasted 60 min, in order to evaluate the potential immediate effects of the tested compounds on P-gp activity as a result of a direct activation of the pump. P-gp activity was evaluated through the ratio between the mean intracellular RHO 123 fluorescence intensity (MFI) obtained under P-gp inhibition (IA) and the MFI observed in normal conditions (NA, in the absence of P-gp inhibition) (Equation (1)), and expressed as percentage of control cells (0 μM chiral ATxs).

**Figure 5:**
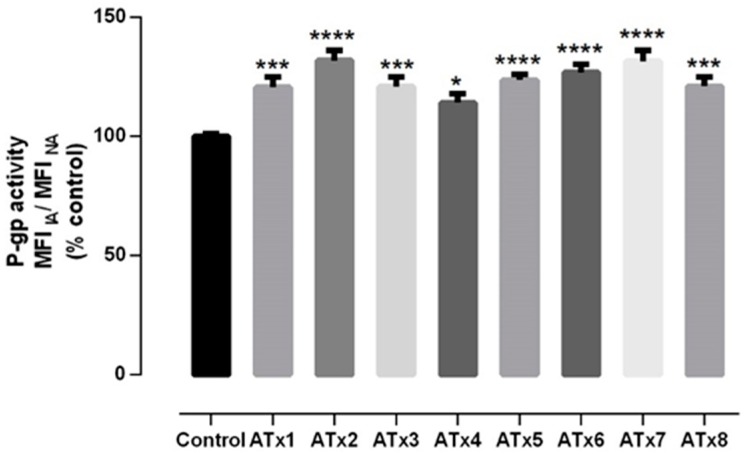
P-gp activity evaluated through the RHO 123 accumulation in the presence of chiral ATxs **1**–**8** (20 µM) during the RHO 123 accumulation phase. Results are presented as mean ± SEM from five independent experiments (performed in triplicate). Statistical comparisons were estimated using One-way ANOVA, followed by Tukey’s multiple comparisons test (* *p* < 0.05; *** *p* < 0.001; **** *p* < 0.0001 vs. control (0 μM)).

A higher P-gp activity, originate a higher ratio from a smaller MFI NA, as the dye is being pumped out of the cells during the accumulation phase. As observed in Figure 5, all the tested compounds **1**–**8** induced a significant increase in P-gp activity, when compared to control cells (P-gp activity significantly increased to 121, 132, 121, 114, 124, 127, 131 and 121% for ATx **1** (+), ATx **2** (−), ATx **3** (+)**,** ATx **4** (−), ATx **5** (+), ATx **6** (−), ATx **7** (+) and ATx **8** (−), respectively). From the tested compounds, ATx **2** (−) and ATx **7** (+) were the most efficient P-gp activators, as exposed by the increased RHO 123 accumulation ratio, that resulted from an decreased RHO 123 intracellular accumulation.

**Figure 6:**
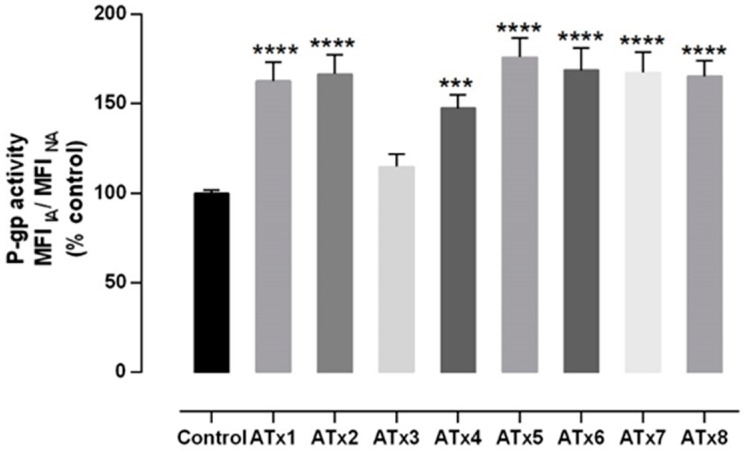
P-gp activity evaluated through the RHO 123 accumulation in Caco-2 cells exposed to chiral thioxanthones (20 µM) for 24 h. Results are presented as mean ± SEM from four independent experiments (performed in duplicate). Statistical comparisons were estimated using One-way ANOVA, followed by Tukey’s multiple comparisons test (*** *p* < 0.001; **** *p* < 0.0001 vs. control (0 μM)).

In the second protocol, RHO 123 accumulation was evaluated in Caco-2 cells pre-exposed to the tested chiral thioxanthones (20 μM) for 24 h. With this second approach, it was possible to assess whether the increased P-gp expression induced by the chiral thioxanthones is accompanied by the matching increases in P-gp activity, given that an increased protein expression does not always mean an increased transport activity [31,59,60,61]. The obtained results (Figure 6) demonstrated a significant increase in P-gp activity for seven of the tested compounds, namely ATx **1** (+), ATx **2** (−) ATx **4** (−), ATx **5** (+), ATx **6** (−), ATx **7** (+) and ATx **8** (−). In fact, P-gp activity increased to 163, 167, 147, 176, 169, 167 and 165% for ATx **1** (+), ATx **2** (−), ATx **4** (−), ATx **5** (+), ATx **6** (−), ATx **7** (+) and ATx **8** (−), respectively. For ATx **3** (+), no significant increase in P-gp activity was observed when compared to control cells (P-gp activity increased only to 115%).

### 2.5. Determination of ATPase Activity

The effect of chiral ATxs **1**–**8** on the ATPase activity of P-gp was evaluated through the detection and quantification of the unmetabolized ATP, as a luciferase-generated luminescent signal, in human P-gp-enriched membranes. P-gp-dependent decreases in luminescence reflect ATP consumption by P-gp and, consequently, a decrease in the obtained signal reflects the presence of a compound that stimulates P-gp ATPase activity.

**Figure 7:**
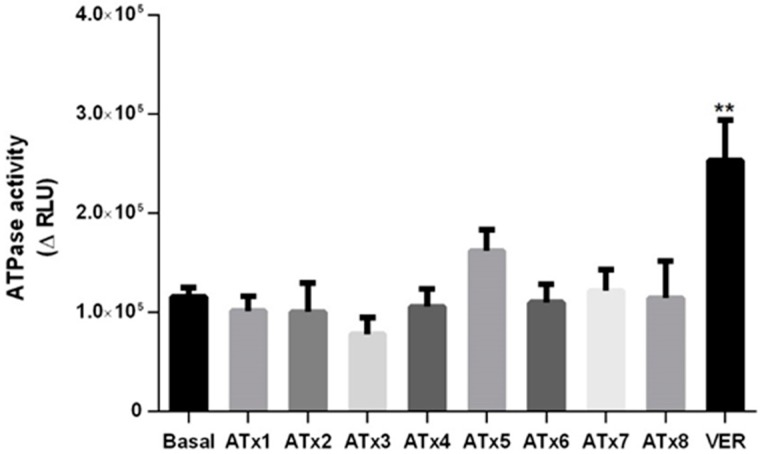
P-gp ATPase activity in the presence of chiral ATxs **1**–**8**. Results are expressed as mean ± SEM from three independent experiments (performed in triplicate). Statistical comparisons were estimated using One-way ANOVA, followed by Tukey’s multiple comparisons test (** *p* < 0.01 vs. basal P-gp ATPase activity).

In the present study, Na_3_VO_4_ (sodium orthovanadate) was used as a selective P-gp inhibitor and, thus, samples treated with Na_3_VO_4_ have no P-gp ATPase activity. Consequently, the difference in luminescent signal between Na_3_VO_4_-treated samples and untreated samples (ΔRLU_basal_) reflects the basal P-gp ATPase activity, whereas the difference in luminescent signal between Na_3_VO_4_-treated samples and samples treated with the test compound (TC) (ΔRLU_TC_) represents the P-gp ATPase activity in the presence of the test compound. Therefore, the test compound can be a stimulator of P-gp ATPase activity if ΔRLU_TC_ > ΔRLU_basal_; can be an inhibitor of P-gp ATPase activity if ΔRLU_TC_ < ΔRLU_basal_; or can have no effect on P-gp ATPase activity if ΔRLU_TC_ = ΔRLU_basal_. Verapamil (Ver) was used in the present assay as a positive control since it is a substrate for P-gp-mediated transport, thus stimulating P-gp ATPase activity. The results for the eight tested compounds and Ver are shown in Figure 7, as well as the basal P-gp ATPase activity. None of the tested compounds (ATx’s **1**–**8**) caused a significant change in the ΔRLU when comparing to the basal P-gp ATPase activity, demonstrating not being substrates for this efflux pump.

## 3. Discussion

All chiral aminated thioxanthones, ATxs **1**–**8**, were synthesized in enantiomerically pure form in order to study the effect of the stereochemistry in P-gp modulation. The reaction conditions used in the Ullmann cross-coupling reaction between a suitable functionalized thioxanthone with enantiomerically pure building blocks allowed a successful synthesis of the desired chiral thioxanthones in moderate yields. The purification process applied allowed us to obtain the desired compounds (ATxs **1**–**8**) with a purity of at least 95% and e.e. higher than 99%. Sufficient amounts were obtained to proceed with the evaluation of the biological activity. Structure elucidation of ATxs **1**–**8** was confirmed by spectroscopic methods. The e.e. values obtained by chiral HPLC evaluation as well as the absolute values of specific rotation confirmed that no racemization occurred under these reaction conditions.

Caco-2 cells express P-gp at levels similar to those found in normal human jejunum [62] and they were already validated as a suitable in vitro model for the evaluation of P-gp modulation [30,31,32,52]. Previous studies demonstrated that newly synthetized thioxanthone derivatives significantly increased both P-gp expression and activity in Caco-2 cells, 24 h after exposure, being the first report on the ability of such compounds to act as P-gp inducers [30]. Moreover, although it is known that increases in protein expression may not necessarily result in proportional increases in the pump activity [31,60,61], for these compounds, the detected increases in the cell surface P-gp expression (since the UIC2 monoclonal antibody used in the experiments recognizes an external P-gp epitope) were accompanied by similar increases in its transport activity [30]. An important aspect to note among the obtained data was the ability of all the tested chiral thioxanthones to rapidly and significantly increase P-gp activity, without interfering with its expression, an effect compatible with a P-gp activation phenomenon [30].

In the present study, ATxs **1**–**8** also demonstrated to be P-gp activators since they were able to significantly increase P-gp transport function without interfering with the protein expression levels, as demonstrated by the data obtained in the RHO 123 accumulation assay performed in the presence of the chiral thioxanthones during a short 60 min accumulation phase. Concerning their structure, a chiral center further away of the thioxanthone moiety seems to prejudice the P-gp activation effect in this series of compounds. The RHO 123 accumulation evaluated using this protocol does not reflect a possible contribution of an increased P-gp expression in the increased activity due to the short duration of the contact between the chiral thioxanthones and the cells during the RHO 123 accumulation phase.

Furthermore, and contrarily to what was observed in the previously mentioned study [30], in the present work, the data obtained for the eight tested compounds demonstrated that, although only chiral ATx **2** (−) caused a significant increase in P-gp expression 24 h after exposure, all the tested compounds significantly increased P-gp activity when RHO 123 accumulation was evaluated 24 h after exposure, except for ATx **3** (+). In fact, and as previously mentioned, it is known that increases in protein expression may not necessarily result in proportional increases in pump activity, and that increases in activity can be observed independently of the level of expression. Indeed, although chiral ATxs **1** (+), **4** (−), **5** (+), **6** (−), **7** (+) and **8** (−) were not able to significantly increase P-gp expression 24 h after exposure, a significant increased P-gp activity was observed in Caco-2 cells pre-exposed to the tested compounds for this incubation period. Therefore, since these compounds demonstrated to be P-gp activators (as demonstrated by the significant differences observed in the RHO 123 accumulation assay performed with the compounds present only during the short RHO 123 accumulation phase), the observed increases in the pump activity must result from a direct pump activation, instead of an increased P-gp expression. The observed P-gp activation may be caused by the compound that remains intracellularly, once the cells are pre-exposed to the tested compounds for 24 h, and washed (to remove the tested compounds) prior to the evaluation of P-gp activity. In what concerns to P-gp ATPase activity, in contrast to previous findings [30], no significant differences were observed between the P-gp ATPase activity in the presence of the investigated thioxanthones and the basal P-gp ATPase activity. These ATxs **1**–**8** with a chiral center in the α-position of the aniline moiety seem not to affect the P-gp ATPase activity, and are unlikely to be substrates for P-gp-mediated transport, since P-gp substrates, like verapamil, typically stimulate its ATPase activity.

Although, some examples of enantioselective modulation of P-gp with other class of compounds have been described [54,55,63,64], no studies were reported focusing the enantioselectivity of chiral thioxanthones in modulating P-gp. Comparing the results obtained for the studied enantiomeric pairs (ATxs **1** (+) and **2** (−), ATxs **3** (+) and **4** (−), ATxs **5** (+) and **6** (−), and ATxs **7** (+) and **8** (−)), no significant differences were observed in P-gp activity. In contrast, for the P-gp induction effect for the chiral pair ATxs **1** (+) and **2** (−), significant differences were found. Noteworthy, a chiral center with *R* configuration closer to the thioxanthone moiety and with less hindered substituents seems to be critical for the induction effect in this series of compounds. This result highlights the existence of enantioselectivity in what concerns to induction mechanism, which is not a consequence of a direct interaction with P-gp. Previously, a similar behavior was described for the H1 anti-histaminic cetirizine, which was also ascribe to effects on P-gp expression [54]; the (*R*)-cetirizine upregulates P-gp expression, while (*S*)-cetirizine down-regulates it [54,65]; although, this library of compounds did not display any direct stereoselective modulation of P-gp, as previously observed for other substrates [54,55,63,64].

## 4. Materials and Methods 

### 4.1. General Information

All reagents and solvents were purchased from Sigma Aldrich Co. (St. Louis, MO, USA), and no further purification was used. The amine building blocks **9**–**16** were (*S*)-(+)-amino-1-propanol (**9**), (*R*)-(−)-2-amino-1-propanol (**10**), (*S*)-(+)-1-amino-2-propanol (**11**), (*R*)-(−)-1-amino-2-propanol (**12**), (*S*)-(+)-leucinol (**13**), (*R*)-(−)-leucinol (**14**), (*S*)-(+)-valinol (**15**) and (*R*)-(−)-valinol (**16**). Dulbecco’s modified Eagle’s medium (high glucose), rhodamine 123 (RHO 123), elacridar and neutral red (NR) solution were obtained from Sigma (St. Louis, MO, USA). Reagents used in cell culture, including nonessential amino acids (NEAA), heat inactivated fetal bovine serum (FBS), 0.25% trypsin/1 mM EDTA, antibiotic (10,000 U/mL penicillin, 10,000 μg/mL streptomycin) and human transferrin (4 mg/mL) were purchased from Gibco Laboratories (Lenexa, KS, USA). P-gp monoclonal antibody (clone UIC2) conjugated with with phycoerythrin (PE) was purchased from Abcam (Cambridge, UK). Flow cytometry reagents (sheath fluid, cleaning solution, decontamination solution, extended flow cell clean) were purchased from BD Bioscience (San Jose, California, USA). The Pgp-Glo™ assay system was purchased from Promega Corporation (Madison, WI, USA). All the reagents used were of analytical grade or of the highest grade available. Reactions were performed in a muffle for 48 h at 100 °C.

Flash column chromatography using silica gel 60 (0.040–0.063 mm, Merck, Darmstadt, Germany), flash cartridge chromatography (GraceResolv^®^, Grace Company, Deerfield, IL, USA), and Discovery^®^ DSC-SCX SPE cationic exchange cartridge (Grace Company, Deerfield, IL, USA) were used in the purification of the synthesized compounds. Melting points (m.p.) were obtained in a Köfler microscope (Santiago, Ostrava, Czech Republic) and are uncorrected. Infrared (IR) spectra were obtained in KBr microplates on a Nicolet iS10 Fourier transform infrared spectroscopy spectrometer from Thermo Scientific (Waltham, MA, USA) with a Smart OMNI-Transmisson accessory (Software OMNIC 8.3, Waltham, MA, USA). Optical rotation measurements were carried out on a Polartronic Universal polarimeter (Bellingham + Stanley Ltd., Tunbridge Wells, Kent, UK). NMR spectra were recorded at the University of Aveiro, Department of Chemistry in CDCl_3_ (Deutero GmbH, Ely, UK) at room temperature on an Avance 300 spectrometer (300.13 MHz for ^1^H and 75.47 MHz for ^13^C, Bruker, Bruker Biosciences Corporation, Billerica, MA, USA). ^13^C-NMR assignments were made by bidimensional HSQC and HMBC experiments (long-range C, H coupling constants were optimized to 7 and 1 Hz) or by comparison with the assignments of similar molecules.

HPLC analysis were performed on a Finnigan Surveyor (Thermo Electron Corporation, Cleveland, OH, USA) equipped with an autosampler (AutoSampler Plus) and a TSP UV8000LP diode array detector (Thermo Electron Corporation). The treatment of the chromatographic data was performed using the Xcalibur^®^2.0 SUR1 software (Thermo Electron Corporation). For purity evaluation, the chromatographic separation was carried out on 250 × 4.6 mm i.d. FortisBIO C18 column (5 μm) with the mobile phase methanol:water:trimethylamine (80:20:0.5 *v*/*v*/*v*). The mobile phase was prepared in a volume/volume relation and degassed in an ultrasonic bath for 15 min before use. The flow rate was 1.0 mL/min. Aliquots of 20 µL of each synthesized chiral thioxanthone (ATxs **1**–**8**) at the concentration of 20 µg/mL were injected. For e.e. evaluation, the chromatographic column used was a 250 × 4.6 mm i.d. Lux^®^ Amylose-1 (5 μm) from Phenomenex (Torrance, CA, USA). Analyses were performed in isocratic mode, at 22 ± 2 °C, in normal phase, using a mixture of *n*-hexane:ethanol (70:30 *v*/*v*) as mobile phase, or polar organic elution mode, using methanol or acetonitrile as mobile phase. The flow rate used was 0.5 mL/min. The UV detection was set at a wavelength of 254 nm and 20 μL of sample were injected in triplicate. The e.e. was determined by the relative percentages of the peak areas according to [e.e. (%) = 100 × ([*R*] − [*S*]/([*R*] + [*S*]) or 100 × ([*S*] − [*R*]/([*S*] + [*R*]), where [*S*] and [*R*] are the peak areas of each enantiomer.

### 4.2. Synthesis of Chiral Thioxanthones (ATxs ***1***–***8***)

1-Chloro-4-propoxy-9*H*-thioxanthen-9-one (Tx, 450 mg, 1.48 mmol) and a suitable chiral amino alcohol (**9***–***16**, 1.72 mmol) were dissolved in methanol (30 mL) and CuI (0.15 mmol) and K_2_CO_3_ (1.92 mmol) were added. The reaction mixture was heated at 100 °C in a muffle furnace for 48 h. After the completion of the reaction, the crude material was filtrated, washed with dichloromethane, and the organic solvents were evaporated under reduced pressure. Then, the obtained solid was dissolved in 50 mL of dichloromethane and extracted with HCl 1 M (3 × 50 mL). The aqueous layer was basified with NaOH 20% and extracted with dichloromethane (3 × 100 mL). The organic layers were gathered, washed with water (3 × 50 mL), dried over anhydrous sodium sulphate and the solvent was evaporated under reduced pressure. Then, a solid phase extraction using a cation exchange cartridge Discovery^®^ DSC-SCX was applied to further purify the extracted material. First, an activation of the cartridge with dichloromethane (50 mL) was performed followed by loading the cartridge with the sample (previously incorporated in silica). Then, elution was carried out with the following solvents: dichloromethane, a mixture of dichloromethane/methanol 5:5 (*v*/*v*), methanol 100% and NH_3_ 2% in methanol. The fractions containing the chiral ATxs were gathered and the solvent was evaporated under reduced pressure. A flash column chromatography with *n*-hexane/ethyl acetate in gradient and crystallization from chloroform and petroleum ether (4:1) were also performed to obtain pure compounds.

*(S)-1-((1-Hydroxypropan-2-yl)amino)-4-propoxy-9H-thioxanthen-9-one* (ATx **1** (+)). Yield: 1.97%. m.p.: 116–118 °C (dec.); ${\left[\alpha \right]}_{D}^{25}$ +112° (c = 3.4 × 10^−3^ g/mL in dichloromethane). IR (KBr) v_max_: 3419, 3270, 2962, 2925, 2873, 2359, 2341, 1618, 1568, 1507, 1435, 1293, 1269, 1252, 1225, 746 cm^−1^. ^1^H-NMR (300.13 MHz, CDCl_3_): δ: 8.50 (1H, d, *J* = 8.0 Hz, H-8), δ: 7.56 (2H, m, H-5 and H-6), δ: 7.43 (1H, m, H-7), δ: 7.13 (1H, d, *J* = 9.0 Hz, H-3), δ: 6.85 (1H, d, *J* = 9.0 Hz, H-2), δ: 4.03 (2H, t, *J* = 6.5 Hz, H-a), δ: 3.81 (3H, m, H-1′ and CH_2_OH), δ: 1.90 (2H, m, H-b), δ: 1.32 (3H, d, *J* = 6.2 Hz, H-2′), δ: 1.12 (3H, t, *J* = 7.4 Hz, H-c). ^13^C-NMR (75.47 MHz, CDCl_3_): δ: 183.55 (C-9), 146.50 (C-4), 144.02 (C-1), 136.85 (C-6), 131.92 (C-8a), 129.85 (C-4a), 129.24 (C-9a), 126.08 (C-8), 125.98 (C-7), 125.20 (C-5), 119.12 (C-3), 114.10 (C-10a), 109.80 (C-2), 72.48 (C-a), 65.96 (C-CH_2_OH), 51.91 (C-1′), 22.66 (C-b), 16.86 (C-2′), 10.71 (C-c). e.e. > 99% (HPLC; column: Lux^®^ Amylose-1 (250 × 4.6 mm i.d., 5 μm), Mobile phase: *n*-hexane:ethanol (70:30 *v*/*v*), 0.5 mL/min, λ_max_ 254 nm).

*(R)-1-((1-Hydroxypropan-2-yl)amino)-4-propoxy-9H-thioxanthen-9-one* (ATx **2** (−)). Yield: 5.91%. m.p.: 118–120 °C (dec.); ${\left[\alpha \right]}_{D}^{25}$ −112° (c = 3.4 × 10^−3^ g/mL in dichloromethane). IR (KBr) v_max_: 3447, 3282, 2963, 2927, 2874, 1611, 1595, 1569, 1506, 1436, 1290, 1270, 1252, 1226, 1072, 795, 746 cm^−1^. ^1^H-NMR (300.13 MHz, CDCl_3_): δ: 8.50 (1H, d, *J* = 8.1 Hz, H-8), δ: 7.56 (2H, m, H-5 and H-6), δ: 7.42 (1H, m, H-7), δ: 7.13 (1H, d, *J* = 9.1 Hz, H-3), δ: 6.82 (1H, d, *J* = 9.1 Hz, H-2), δ: 4.04 (2H, t, *J* = 6.5 Hz, H-a), δ: 3.78 (3H, m, H-1′ and CH_2_OH), δ: 1.90 (2H, m, H-b), δ: 1.33 (3H, d, *J* = 6.3 Hz, H-2′), δ: 1.11 (3H, m, H-c). ^13^C-NMR(75.47 MHz, CDCl_3_): δ: 183.56 (C-9), 146.80 (C-4), 144.00 (C-1), 136.82 (C-6), 131.87 (C-8a), 129.91 (C-4a), 129.22 (C-9a), 126.05 (C-8), 125.97 (C-7), 125.20 (C-5), 119.35 (C-3), 114.00 (C-10a), 109.02 (C-2), 72.52 (C-a), 66.04 (C-CH_2_OH), 51.55 (C-1′), 22.70 (C-b), 17.00 (C-2′), 10.71 (C-c). e.e. > 99% (HPLC; column: Lux^®^ Amylose-1 (250 × 4.6 mm i.d., 5 μm), Mobile phase: *n*-hexane:ethanol (70:30 *v*/*v*), 0.5 mL/min, λ_max_ 254 nm).

*(S)-1-((2-Hydroxypropyl)amino)-4-propoxy-9H-thioxanthen-9-one* (ATx **3** (*+*)). Yield: 7.86%. m.p.: 149–152 °C (dec.); ${\left[\alpha \right]}_{D}^{25}$ −12° (c = 6.67 × 10^−3^ g/mL in dichloromethane). IR (KBr) v_max_: 3460, 3320, 2965, 2933, 2875, 1607, 1566, 1556, 1502, 1450, 1434, 1286, 1269, 1249, 1227, 1158, 1133, 1060, 744 cm^−1^. ^1^H-NMR(300.13 MHz, CDCl_3_): δ: 8.52 (1H, d, *J* = 8.1 Hz, H-8), δ: 7.56 (2H, m, H-5 and H-6), δ: 7.42 (1H, m, H-7), δ: 7.13 (1H, d, *J* = 9.0 Hz, H-3), δ: 6.78 (1H, d, *J* = 9.0 Hz, H-2), δ: 4.26 (1H, m, H-2′), δ: 4.03 (2H, t, *J* = 6.5 Hz, H-a), δ: 3.30 (2H, m, H-1′), δ: 1.90 (2H, m, H-b), ^TM^: 1.35 (3H, d, *J* = 6.3, CH3), δ: 1.12 (3H, t, *J* = 7.4, H-c). ^13^C-NMR (75.47 MHz, CDCl_3_): δ: 183.46 (C-9), 146.18 (C-4), 144.01 (C-1), 136.98 (C-6), 133.27 (C-8a), 129.54 (C-4a), 129.43 (C-9a), 127.19 (C-8), 126.32 (C-7), 126.10 (C-5), 117.94 (C-3), 115.36 (C-10a), 111.83 (C-2), 72.31 (C-a), 65.58 (C-2′), 54.12 (C-1′), 22.74 (C-b), 20.97 (C-CH3), 14.15 (C-c). e.e. > 99% (HPLC; column: Lux^®^ Amylose-1 (250 × 4.6 mm i.d., 5 μm), Mobile phase: methanol, 0.5 mL/min, λ_max_ 254 nm).

*(R)-1-((2-Hydroxypropyl)amino)-4-propoxy-9H-thioxanthen-9-one* (ATx **4** (*−*)). Yield: 23.62%. m.p.: 145–150 °C (dec.); ${\left[\alpha \right]}_{D}^{25}$ +12° (c = 6.67 × 10^−3^ g/mL in dichloromethane). IR (KBr) v_max_: 3460, 3323, 2966, 2932, 2875, 1606, 1566, 1556, 1501, 1451, 1434, 1328, 1286, 1270, 1249, 1227, 1158, 1133, 1102, 1060, 972, 744 cm^−^^1^. ^1^H-NMR (300.13 MHz, CDCl_3_): δ: 8.51 (1H, d, *J* = 8.0 Hz, H-8), δ: 7.50 (2H, m, H-5 and H-6), δ: 7.41 (1H, m, H-7), δ: 7.12 (1H, d, *J* = 9.0 Hz, H-3), δ: 6.73 (1H, d, *J* = 9.0 Hz, H-2), δ: 4.22 (1H, m, H-2′), δ: 4.03 (2H, t, *J* = 6.4 Hz, H-a), δ: 3.29 (2H, m, H-1′), δ: 1.90 (2H, m, H-b), δ: 1.35 (3H, d, *J* = 6.3, CH_3_), δ: 1.12 (3H, t, *J* = 7.4, H-c). ^13^C-NMR (75.47 MHz, CDCl_3_): δ: 183.46 (C-9), 147.48 (C-4), 143.91 (C-1), 136.81 (C-6), 131.83 (C-8a), 129.92 (C-4a), 129.85 (C-9a), 129.26 (C-8), 126.02 (C-7), 125.95 (C-5), 119.25 (C-3), 113.95 (C-10a), 108.42 (C-2), 72.47 (C-a), 66.11 (C-2′), 52.07 (C-1′), 22.85 (C-b), 21.02 (C-CH_3_), 10.71 (C-c). e.e. > 99% (HPLC; column: Lux^®^ Amylose-1 (250 × 4.6 mm i.d., 5 μm), Mobile phase: methanol, 0.5 mL/min, λ_max_ 254 nm).

*(S)-1-((1-Hydroxy-4-methylpentan-2-yl)amino)-4-propoxy-9H-thioxanthen-9-one* (ATx **5** (*+*)). Yield: 19.59%. m.p.: 82–85 °C (dec.); ${\left[\alpha \right]}_{D}^{25}$ −68° (c = 6.75 × 10^−3^ g/mL in dichloromethane). IR (KBr) v_max_: 3457, 3290, 2955, 2923, 2870, 1613, 1595, 1566, 1505, 1464, 1435, 1386, 1265, 1224, 1107, 1067, 1018, 745 cm^−1^. ^1^H-NMR (300.13 MHz, CDCl_3_): δ: 8.51 (1H, d, *J* = 8.1 Hz, H-8), δ: 7.57 (2H, m, H-5 and H-6), δ: 7.42 (1H, m, H-7), δ: 7.13 (1H, d, *J* = 9.0 Hz, H-3), δ: 6.93 (1H, d, *J* = 9.0 Hz, H-2), δ: 4.04 (2H, t, *J* = 6.5 Hz, H-a), δ: 3.88 (1H, dd, *J* = 10.1 Hz, H-1′), δ: 3.71 (2H, m, CH_2_OH), δ: 1.85 (4H, m, H-b), δ:1.78 (1H, m, H-3′), δ: 1.57 (1H, t, *J* = 6.9 Hz, H-2′), δ: 1.12 (3H, t, *J* = 7.4 Hz, H-c), δ: 0.94 (3H, d, *J* = 6.5 Hz, H-4′a), δ: 0.89 (3H, d, *J* = 6.5 Hz, H-4′b). ^13^C-NMR (75.47 MHz, CDCl_3_): δ: 183.61 (C-9), 145.00 (C-4), 142.80 (C-1), 136.7 (C-6), 131.98 (C-8a), 129.97 (C-4a), 129.83 (C-9a), 129.28 (C-8), 126.11 (C-7), 126.00 (C-5), 119.05 (C-3), 114.43 (C-10a), 111.02 (C-2), 72.45 (C-a), 64.62 (C-1′), 55.01 (C-CH_2_OH), 40.44 (C-2′), 24.90 (C-3′), 22.86 (C-4′a), 22.83 (C-4′b), 22.61 (C-b), 10.71 (C-c). e.e. > 99% (HPLC; column: Lux^®^ Amylose-1 (250 × 4.6 mm i.d., 5 μm), Mobile phase: acetonitrile, 0.5 mL/min, λ_max_ 254 nm).

*(R)-1-((1-Hydroxy-4-methylpentan-2-yl)amino)-4-propoxy-9H-thioxanthen-9-one* (ATx **6** (*−*)). Yield: 10.40%. m.p.: 80–82 °C (dec.); ${\left[\alpha \right]}_{D}^{25}$ +56° (c = 6.75 × 10^−3^ g/mL in dichloromethane). IR (KBr) v_max_: 3459, 3291, 2954, 2925, 2871, 1613, 1595, 1566, 1505, 1480, 1435, 1386, 1266, 1224, 1108, 1072, 1018, 746 cm^−1^. ^1^H-NMR (300.13 MHz, CDCl_3_): δ: 8.51 (1H, d, *J* = 8.1 Hz, H-8), δ: 7.55 (2H, m, H-5 and H-6), δ: 7.42 (1H, m, H-7), δ: 7.12 (1H, d, *J* = 9.1 Hz, H-3), δ: 6.86 (1H, d, *J* = 9.1 Hz, H-2), δ: 4.03 (2H, t, *J* = 6.5 Hz, H-a), δ: 3.85 (1H, dd, *J* = 10.5 , H-1′), δ: 3.72 (2H, m, CH_2_OH), δ: 1.83 (4H, m, H-b), δ: 1.76 (1H, m, H-3′), δ: 1.57 (1H, t, *J* = 6.9 Hz, H-3′), δ: 1.12 (3H, t, *J* = 7.4 Hz, H-c), δ: 0.96 (3H, d, *J* = 6.5 Hz, H-4′a), δ: 0.89 (3H, d, *J* = 6.5 Hz, H-4′b). ^13^C-NMR (75.47 MHz, CDCl_3_): δ: 183.61 (C-9), 145.00 (C-4), 142.80 (C-1), 136.82 (C-6), 131.87 (C-8a), 129.93 (C-4a), 129.24 (C-9a), 128.80 (C-8), 126.03 (C-7), 125.97 (C-5), 119.42 (C-3), 113.94 (C-10a), 108.80 (C-2), 72.49 (C-a), 64.87 (C-1′), 40.71 (C-2′), 24.92 (C-3′), 22.96 (C-4′a), 22.86 (C-4′b), 22.55 (C-b), 10.71 (C-c). e.e. > 99% (HPLC; column: Lux^®^ Amylose-1 (250 × 4.6 mm i.d., 5 μm), Mobile phase: acetonitrile, 0.5 mL/min, λ_max_ 254 nm).

*(S)-1-((1-Hydroxy-3-methylbutan-2-yl)amino)-4-propoxy-9H-thioxanthen-9-one* (ATx **7** (+)). Yield: 10.12%. m.p.: 98–102 °C (dec.); ${\left[\alpha \right]}_{D}^{25}$ −26° (c = 6.80 × 10^−3^ g/mL in dichloromethane). IR (KBr) v_max_: 3385, 3256, 2960, 2926, 2873, 2360, 1614, 1569, 1514, 1463, 1434, 1386, 1329, 1267, 1249, 1225, 1165, 1070, 1021, 744 cm^−1^. ^1^H-NMR (300.13 MHz, CDCl_3_): δ: 8.54 (1H, d, *J* = 8.0 Hz, H-8), δ: 7.59 (2H, m, H-5 and H-6), δ: 7.45 (1H, m, H-7), δ: 7.14 (1H, d, *J* = 9.1 Hz, H-3), δ: 7.07 (1H, d, *J* = 9.1 Hz, H-2), δ: 4.06 (2H, t, *J* = 6.4 Hz, H-a), δ: 3.86 (2H, m, CH_2_OH),δ: 3.53 (1H, m, H-1′), δ: 2.08 (1H, m, H-2′), δ: 1.91 (2H, m, H-b), δ: 1.05 (3H, d, *J* = 6.9 Hz, H-3′a), δ: 0.87 (3H, d, *J* = 6.9 Hz, H-3′b), δ: 1.12 (3H, t, m, H-c). ^13^C-NMR (75.47 MHz, CDCl_3_): δ: 183.43 (C-9), 145.0 (C-4), 142.8 (C-1), 136.43 (C-6), 131.66 (C-8a), 129.53 (C-4a), 129.03 (C-9a), 128.84 (C-8), 125.70 (C-7), 125.51 (C-5), 117.82 (C-3), 114.71 (C-10a), 111.6 (C-2), 71.80 (C-a), 63.93 (C-1′), 61.56 (C-CH_2_OH), 29.15 (C-2′), 22.21 (C-b), 18.53 (C-3′a), 18.41 (C-3′b), 10.14 (C-c). e.e. > 99% (HPLC; column: Lux^®^ Amylose-1 (250 × 4.6 mm i.d., 5 μm), Mobile phase: acetonitrile, 0.5 mL/min, λ_max_ 254 nm).

*(R)-1-((1-Hydroxy-3-methylbutan-2-yl)amino)-4-propoxy-9H-thioxanthen-9-one* (ATx **8** (−)). Yield: 10.24%. m.p.: 98–104 °C (dec.); ${\left[\alpha \right]}_{D}^{25}$ +26° (c = 6.80 × 10^−3^ g/mL in dichloromethane). IR (KBr) v_max_: 3423, 3272, 2959, 2925, 2872, 1611, 1570, 1511, 1463, 1435, 1385, 1251, 1226, 1161, 1068, 1022, 747 cm^−1^.^1^H-NMR(300.13 MHz, CDCl_3_): δ: 8.56 (1H, d, *J* = 8.0 Hz, H-8), δ: 7.64 (2H, m, H-5 and H-6), δ: 7.49 (1H, m, H-7), δ: 7.34 (1H, d, *J* = 8.9 Hz, H-3), δ: 7.17 (1H, d, *J* = 8.9 Hz, H-2), δ: 4.10 (2H, t, *J* = 6.4 Hz, H-a), δ: 3.90 (2H, m, CH_2_OH), δ: 3.50 (1H, m, H-1′), δ: 2.10 (1H, m, H-2′), δ: 1.91 (2H, m, H-b), δ: 1.02 (3H, d, *J* = 6.9 Hz, H-3′a), δ: 0.98 (3H, d, *J* = 6.9 Hz, H-3′b), δ: 1.14 (3H, t, m, H-c); ^13^C-NMR (75.47 MHz, CDCl_3_): δ: 183.65 (C-9), 147.74 (C-4), 141.53 (C-1), 137.16 (C-6), 132.60 (C-8a), 130.43 (C-4a), 129.52 (C-9a), 129.21 (C-8), 126.52 (C-7), 126.19 (C-5), 117.12 (C-3), 116.69 (C-10a), 115.38 (C-2), 72.19 (C-a), 67.02 (C-1′), 61.17 (CH_2_OH), 28.87 (C-2′), 22.65 (C-b), 19.31 (C-3′a), 18.58 (C-3′b), 10.69 (C-c). e.e. > 99% (HPLC; column: Lux^®^ Amylose-1 (250 × 4.6 mm i.d., 5 μm), Mobile phase: acetonitrile, 0.5 mL/min, λ_max_ 254 nm).

### 4.3. Caco-2 Cell Culture

For the in vitro assays, the Caco-2 cell model was used, which derived from human colorectal adenocarcinoma. The cells were routinely cultured in 75 cm^2^ flasks and maintained in a 5% CO_2_–95% air atmosphere, at 37 °C. Dulbecco’s modified Eagle’s medium (DMEM) supplemented with 10% heat-inactivated fetal bovine serum (FBS), 100 μM nonessential amino acids (NEAA), 100 U/mL penicillin, 100 µg/mL streptomycin and 6 μg/mL transferrin was used and changed every 2 to 3 days. Cultures were passaged weekly by trypsinization (0.25% trypsin/1 mM EDTA). For all the experiments, the cells were taken between the 27th and 36th passages. The cells were always seeded at a density of 60,000 cells/cm^2^ and used 3 days after seeding, when confluence was reached. For the cytotoxicity assays the cells were seeded in 96-well plates, while for the evaluation of P-gp expression and P-gp transport activity, the cells were seeded in 12-well plates.

### 4.4. Compounds Cytotoxicity Assays

The cytotoxicity of the chiral ATxs **1**–**8** (0–50 μM) was accessed by the NR uptake assay, 24 h after exposure. The NR uptake assay provides a quantitative estimation of the number of viable cells in a culture based on the ability of viable cells to incorporate and bind the supravital dye NR in the lysosomes [30]. For that purpose, the cells were seeded onto 96-well plates and exposed to the eight tested ATxs **1**–**8** (0–50 μM) in fresh cell culture medium. After a 24 h incubation period, the cells were incubated with NR (40 μg/mL in cell culture medium) at 37 °C, in a humidified, 5% CO_2_–95% air atmosphere, for 60 min. The cell culture was then removed, the dye absorbed only by viable cells extracted (with absolute ethyl alcohol/distilled water (1:1 *v*/*v*) containing 5% acetic acid) and the absorbance measured at 540 nm in a multiwell plate reader (BioTek Synergy™ HT, BioTek, Winooski, VT, USA). At least five independent experiments were performed, in triplicate, and the percentage of NR uptake relative to that of the control cells was used as the cytotoxicity measure.

### 4.5. Flow Cytometry Analysis of P-gp Expression

P-gp expression was evaluated by flow cytometry using a P-gp monoclonal antibody [UIC2] conjugated with phycoerythrin (PE) [30]. For that purpose, Caco-2 cells were seeded onto 12-well plates, and exposed, three days after seeding, to the eight tested ATxs **1**–**8** (20 μM) in fresh cell culture medium. After 24 h of incubation, the cells were washed with HBSS buffer and harvested by trypsinization (0.25% trypsin/1 mM EDTA) to obtain a cellular suspension. After centrifugation (300× *g*, for 10 min, at 4 °C), the cells were suspended in HBSS buffer containing the P-gp antibody, being the antibody dilution selected accordingly to the manufacturer’s instructions for flow cytometry. After a 30 min incubation, at 37 °C in the dark, with the UIC2 antibody, the cells were washed twice with HBSS containing 10% heat-inactivated FBS, centrifuged (300× *g* for 10 min), suspended in ice-cold HBSS buffer and kept on ice until analysis. The fluorescence measurements of isolated cells were performed with a flow cytometer (BD AccuriTM C6, BD Biosciences, San Jose, California, USA), being the fluorescence of the UIC2-PE antibody measured by a 585 ± 40 nm band-pass filter (FL2). The logarithmic fluorescence was recorded and displayed as a single parameter histogram, and based on the acquisition of data for 20,000 cells. The parameter used for comparison was the mean of fluorescence intensity (MFI) [calculated as percentage of control (0 μM)]. In order to detect a possible contribution from cells autofluorescence to the analyzed fluorescence signals, non-labelled cells (with or without chiral thioxanthones) were also analyzed in each experiment by a 585 ± 40 nm band-pass filter (FL2). Five independent experiments were performed in duplicate.

### 4.6. Evaluation of P-gp Transport Activity 

The effect of the tested compounds on P-gp transport activity was accessed by flow cytometry using RHO 123 (2 µM) as a P-gp fluorescent substrate. Two different approaches were tested: a RHO 123 accumulation assay in the presence (IA) and absence (NA) of elacridar (10 μM), a known P-gp inhibitor, and with or without simultaneous exposure to ATxs **1**–**8** (20 μM) only during the RHO 123 accumulation phase; a RHO 123 accumulation assay, in the presence or absence of elacridar (10 μM), with or without pre-exposure of Caco-2 cells to ATxs **1**–**8** (20 μM) for 24 h.

#### 4.6.1. RHO 123 Efflux Assay in the Presence of ATxs **1**–**8**

For this purpose, the cells were seeded onto 75 cm^2^ flasks and, after reaching confluence, harvested by trypsinization to obtain a cellular suspension, which was then divided into several aliquots of 300,000 cells. After centrifugation (300× *g* for 10 min), the cells were suspended in HBSS containing 10% heat-inactivated FBS and 2 μM RHO 123, with and without 20 μM ATxs **1**–**8**, and incubated at 37 °C, for 60 min, in the presence and absence of elacridar (10 μM). The cells were then washed twice with ice-cold HBSS with 10% heat-inactivated FBS, centrifuged (300× *g* for 10 min at 4 °C) and kept on ice until flow cytometry analysis. The fluorescence measurements of isolated cells were performed as described in the section “Evaluation of P-gp expression”, being the RHO 123 fluorescence measured by a 530 ± 15 nm band-pass filter (FL1). The activity of the efflux pump was accessed by the RHO 123 accumulation ratio (Equation (1)) and expressed as percentage of control cells (0 μM ATXs):

(1) $$\mathrm{RHO}\;123\;\mathrm{accumuation}=\;\frac{\mathrm{MFI}\;\mathrm{of}\;\mathrm{RHO}\;123\;\mathrm{accumuation}\;\mathrm{under}\;\mathrm{inhibition}\;\left(\mathrm{IA}\right)}{\mathrm{MIF}\;\mathrm{of}\;\mathrm{RHO}\;123\;\mathrm{normal}\;\mathrm{accumuation}\;\left(\mathrm{NA}\right)}$$

P-gp activity was assessed by ratio between the amount of RHO 123 accumulated under P-gp inhibition (with 10 μM elacridar) and the amount of RHO 123 accumulated in the absence of the P-gp inhibitor.

An increase in P-gp activity results in an increased RHO 123 efflux from the cells, which results in a decrease in the intracellular fluorescence intensity (decreased intracellular RHO 123 content). Therefore, a higher RHO 123 accumulation ratio (Equation (1)) is a consequence of a smaller MFI NA, which results from a higher P-gp activity since the dye is being effluxed out of the cells during the accumulation phase. Five independent experiments were performed in triplicate.

#### 4.6.2. RHO 123 Efflux Assay in Caco-2 Cells Pre-Expose to ATxs **1**–**8** for 24 h

Caco-2 cells, seeded onto 12-well plates, were exposed to chiral thioxanthones (20 μM), in fresh cell culture medium, for 24 h, prior to the evaluation of P-gp activity. After exposure, the cells were harvested by trypsinization to obtain a cell suspension, being the cells of each well divided into two aliquots. One aliquot was submitted to a RHO 123 accumulation phase under inhibited conditions (RHO 123 accumulation performed in IA conditions) and the second aliquot was submitted to a RHO 123 accumulation phase performed under normal conditions (RHO 123 accumulation performed in NA conditions). For IA accumulation, the cells were centrifuged (300× *g* for 10 min) and suspended in HBSS buffer (pH 7.4) containing 10% heat-inactivated FBS, 2 µM RHO 123 and the P-gp inhibitor elacridar (10.0 µM), while for the NA accumulation a similar incubation was performed but in the absence of the P-gp inhibitor. In both cases, the cells were incubated with the P-gp substrate at 37 °C for 60 min to allow RHO 123 accumulation. After this accumulation period, the cells were washed twice with ice-cold HBSS buffer containing 10% heat-inactivated FBS and suspended in ice-cold HBSS buffer immediately before analysis. The evaluation of RHO 123 intracellular content was performed as described in the “Flow cytometry analysis of P-gp expression” section, being the RHO 123 fluorescence measured by a 530 ± 15 nm band-pass filter (FL1). The results were calculated according to the ratio defined in Equation (1) and expressed as percentage of control cells. Four independent experiments were performed in duplicate.

### 4.7. Evaluation of P-gp ATPase Activity

The effects of the tested ATxs **1**–**8** on P-gp ATPase activity were evaluated in recombinant human P-gp-enriched membranes, using the luminescent Pgp-Glo™ Assay (Promega Corporation, Madison, WI, USA), according to the manufacturer’s instructions.

Briefly, recombinant human P-gp-enriched membranes (25 μg/well) were incubated with 20 μM chiral thioxanthones or sodium vanadate at 100 μM (positive control for P-gp ATPase activity inhibition) or verapamil at 200 μM (positive control for P-gp ATPase activity stimulation) in assay buffer, and 5 mM MgATP, for exactly 60 min, at 37 °C. After incubation, the reaction was stopped and the remaining ATP detected in a multi-well plate reader (BioTek Synergy™ HT), after a 20 min signal-developing period at room temperature, as a luciferase-generated luminescent signal.

The decreased luminescence of the untreated (NT) incubations relative to Na_3_VO_4_-treated incubations reflects the basal P-gp ATPase activity. The decreased luminescence of incubations performed with the positive control drug verapamil relative to Na_3_VO_4_-treated reactions reflects verapamil-stimulated P-gp ATPase activity. The luminescence of the chiral thioxanthones incubations relative to that of the Na_3_VO_4_-treated reactions reflects the effect, if any, of that compounds on P-gp ATPase activity (i.e., a decrease in luminescence reflects a stimulated P-gp ATPase activity). Three independent experiments were performed in triplicate for this assay and the results expressed as mean ± SEM.

### 4.8. Statistical Analysis

All statistical calculations were performed with the GraphPad Prism version 6.00 for Windows (GraphPad Software, San Diego, CA, USA). Normality of the data distribution was assessed by three different tests (KS normality test, D’Agostino & Pearson omnibus normality test and Shapiro-Wilk normality test). Data obtained from the thioxanthones cytotoxicity assays are expressed as mean ± SEM from at least five independent experiments (performed in triplicate) and the statistical comparisons were estimated using the nonparametric method of Kruskal–Wallis, followed by the Dunn’s post hoc test. Data from the P-gp expression assays are presented as mean ± SEM from five independent experiments (performed in duplicate) and the statistical comparisons were made using One-way ANOVA, followed by the Tukey’s multiple comparisons test. Results from the P-gp transport activity assays performed in the presence of chiral thioxanthones during the RHO 123 accumulation phase are presented as mean ± SEM from five independent experiments (performed in triplicate) and the statistical comparisons were estimated using One-way ANOVA, followed by the Tukey’s multiple comparisons test. Results from the P-gp transport activity assays performed in cells pre-exposed to chiral thioxanthones for 24 h are presented as mean ± SEM from four independent experiments (performed in duplicate) and the statistical comparisons were estimated using One-way ANOVA, followed by the Tukey’s multiple comparisons test. Results from the P-gp ATPase activity is presented as mean ± SEM from three independent experiments (performed in triplicate) and the statistical comparisons were estimated using One-way ANOVA, followed by the Tukey’s multiple comparisons test. In all cases, *p* values lower than 0.05 were considered significant.

## 5. Conclusions

Thioxanthone derivatives and, particularly, aminated thioxanthones, have been characterized as P-gp modulators. Since the pharmacokinetics, efficacy and safety of drugs that are P-gp substrates depend on the level of expression and functionality of P-gp, and can be affected by the chirality of compounds, it is important to better understand the effect that this important property may have in P-gp modulation. Therefore, in order to accomplish the main aim of this study, four enantiomeric pairs of thioxanthones were synthesized through a copper catalyzed Ullman asymmetric coupling reaction that provided the desired chiral thioxanthones with an e.e. > 99%. The assays carried out for the evaluation of the biological activity of the tested chiral ATxs **1**–**8** demonstrated that these compounds have the ability to immediately increase P-gp activity without interfering with its levels of expression and, therefore, can be characterized as P-gp activators. Chiral thioxanthones **2** (−) and **7** (+) demonstrated to be the most efficient P-gp activators, among all the thioxanthones tested.

Comparing the results obtained with both enantiomers of each enantiomeric pair, no significant enantioselectivity was observed for the direct modulation of P-gp. Nevertheless, the introduction of a chiral center close to the thioxanthonic scaffold favored their activation effect. Differences were noted in what concerns the induction effect, which may be related to their mechanism of induction, which deserves to be further explored. However, it is well known that P-gp expression is regulated by multiple signaling pathways, each involving different molecular targets and transcription factors, making the elucidation of the induction mechanism underlying ATX2-mediated P-gp induction a complex task. One of the possible pathways involved in the transcriptional activation of the *MDR1* gene expression involves the activation of the pregnane X receptor (PXR) [4,66], and several anticancer compounds, plant extracts, cholesterol-lowering statins, rifampicin and HIV protease inhibitors were already reported as PXR ligands [67,68,69,70,71,72]. Therefore, the potential enantioselectivity in what concerns to PXR activation deserves to be explored in future studies.
